# Effects of 1-methylcyclopropene, methyl jasmonate and salicylic acid on physicochemical properties and wooliness of nectarine fruit during cold storage

**DOI:** 10.1186/s12870-024-05939-z

**Published:** 2024-12-19

**Authors:** Fatih Sen, Enes Yilmaz, Burhan Ozturk

**Affiliations:** 1 Faculty of Agriculture, Department of Horticulture, Ege University, İzmir, Türkiye; 2 Faculty of Agriculture, Department of Horticulture, Ordu University, Ordu, Türkiye

**Keywords:** Antioxidant, Flesh firmness, Phenolics, Plant growth regulators, Weight loss

## Abstract

**Background:**

Owing to its high perishability, the market life of nectarine fruit is very short. Cold storage is a principal approach to limit post-harvest quality loss in nectarines. The objective of this research was to evaluate the impact of postharvest methyl jasmonate (MeJA), salicylic acid (SA) and 1-methylcyclopropene (1-MCP) on quality properties of nectarine fruit, specifically weight loss, firmness, phenolics and antioxidant activity, following cold storage and subsequent shelf life. Fruit immersed in water were considered as control. The fruit were stored at 0 ± 0.5 °C and 90 ± 5% RH for 56 d, then kept at 20 ± 0.5 °C and 70 ± 5% RH for 2 d in shelf life.

**Results:**

The results demonstrated that single or combined treatments of MeJA, SA and 1-MCP were effective on quality characteristics. During cold storage, fruit treated with MeJA + 1-MCP (3.66%) and SA + 1-MCP (3.54%) exhibited lower weight loss than the control (4.08%). In the final two measurements of storage, the flesh firmness of fruit treated with SA + 1-MCP (54.5 and 54.06 N, respectively) was higher than that of the control. At the end of cold storage, the SA + 1-MCP treatment (17.4%) exhibited higher soluble solids than the control (15.37%) and SA (15.20%) treatments. However, the total phenolics content was found to be higher in nectarine fruit treated with single SA than in the control, as well as in fruit treated with SA + 1-MCP and single 1-MCP. Wooliness in fruit treated with 1-MCP, SA and MeJA was found to be lower than in the control, while fruit had higher acceptance.

**Conclusions:**

As a result, the SA + 1-MCP and MeJA + 1-MCP treatments were more efficacious in retarding the weight and firmness decline of nectarine fruit during storage. Also, it was revealed that 1-MCP, SA and MeJA could be employed as efficacious instruments in nectarine fruit with respect to wooliness and acceptance, which influence consumer preferences.

## Background

Peaches (*Prunus persica* L. Batsch) and nectarines (*Prunus persica* var. nectarina Maxim.) are the third most economically delicious and valuable fruit crops globally, following apples (*Malus* spp.) and pears (*Pyrus* spp.) [1]. Peaches and nectarines are consumed primarily as fresh fruit by consumers. Due to the longer post-harvest life of nectarines, their preservation and export to distant markets is more advantageous than that of peaches. However, as the storage period is prolonged, significant losses are experienced in nectarine fruit, similar to those experienced in many other fresh fruits and vegetables. In recent years, the use of pre-harvest or post-harvest development regulator applications has become a prominent strategy employed to minimize these losses. In particular, the use of growth regulators, such as methyl jasmonate (MeJA), salicylic acid (SA) and 1-methylcyclopropene (1-MCP), has been reported to play a positive role in extending cold storage and shelf life by delaying postharvest quality losses in a number of fruit and vegetable species [2, 3].

In fruits and vegetables, biochemical reactions can be slowed down by delaying ripening using ethylene inhibitors after harvesting. Therefore, in order to prolong the storage and shelf life of climacteric fruits, ethylene-induced ripening should be delayed. The use of ethylene inhibitors after harvest can delay biochemical reactions in fruits and vegetables, thereby extending their storage and shelf life. 1-methylcyclopropene (1-MCP) is the most commonly employed ethylene inhibitor following harvesting [4]. It has been demonstrated that the application of 1-MCP prolongs shelf-life and maintains quality [5].

Jasmonic acid or its methyl ester methyl jasmonate (MeJA), which is naturally present in most plants, can affect numerous physiological and biochemical processes in plants [2]. Due to its synergistic and antagonistic effects with other growth regulators, it can play both a promoting and inhibitory role in plant growth [6]. It has been demonstrated that MeJA can mitigate chilling injury in fruits [7], enhance the accumulation of bioactive compounds and certain fruit quality parameters [2, 8, 9], and prolong storage and shelf life [10–12].

Salicylic acid (SA) is a growth regulator that plays a pioneering role in increasing resistance in the plant defense system against biotic and abiotic stresses. In addition, it can affect plant growth and development by manipulating various metabolic and physiological events in plants. It has been reported that postharvest applications can reduce chilling damage and rot in fruits, as well as delay the loss of nutrient content, fruit firmness, appearance and fruit quality characteristics [13]. Nevertheless, postharvest applications have been documented to delay ripening in fruit species such as cherry, kiwifruit, peach and apple [9].

In the literature, although there are studies on the effects of single applications of growth regulators on nectarine fruit [5, 14], there is no research on how 1-MCP works when combined with MeJA and SA. Therefore, the research question of the study is: Do combined applications of 1-MCP, MeJA and SA have any effect on delaying weight loss and preservation of fruit quality characteristics during storage of nectarine fruit? The study hypothesized that the combined application of growth regulators would better maintain fruit quality characteristics during the shelf life of 2 days [15] following cold storage.

The primary objective of this study was to assess the impact of combined applications with 1-MCP of MeJA and SA on the delay of weight loss and maintenance of fruit quality in the ‘Extreme Sunny’ nectarine cultivar during storage.

## Materials and methods

### Plant materials

The plant material for the study was obtained from a commercial nectarine orchard in the Selçuk district of Izmir, Türkiye. The nectarine fruit were picked from 9-year-old ‘Extreme Sunny’ nectarine trees (*Prunus persica* var. *nectarina* Maxim.) grafted on Garnem rootstock. Nectarine fruit were hand-harvested at the optimal commercial ripeness (h° ∼70 and flesh firmness: ∼65 N). The trees were planted at a distance of 5 m between rows and 3 m above rows, and were subsequently trained in the Catalonian Vase system. Cultural operations, including pruning, tillage, fertilization, disease and pest control, were conducted on a regular basis in the nectarine orchard.

## Methyl jasmonate, salicylic acid and 1-methylcyclopropene treatments

Undamaged, uniformly shaped, and of homogeneous size and color were selected and placed in plastic crates. The fruit were then transferred to the Cold Storage and Packaging Unit at Ege University, Faculty of Agriculture, Department of Horticulture within two hour (h) by refrigerated vehicle [12 ± 1 °C and 80 ± 5% relative humidity (RH)].

Firstly, nectarine fruit were immersed in a 100-ppm sodium hypochlorite solution for two min in order to achieve surface sterilization. Subsequently the fruit were divided into three groups. The first group of fruit was immersed in a solution containing 0.2 mM methyl jasmonate [(MeJA), Merck, Germany] for two min, while the second group was immersed in a solution (20 °C) containing 2 mM salicylic acid [(SA), Merck, Germany]. The third group was immersed in water for two min, serving as the control group. Surfactant [0.04%, Nu-Film-17^®^, (Miller Chemical Corp., USA)] was added to the solution used in all applications.

The fruit were placed in modified atmosphere packaging [MAPs (LifePack, Aypek, Türkiye)]. Nectarine fruit treated with MeJA and SA, and control fruit were divided into two groups, with one group (1-MCP, MeJA + 1-MCP and SA + 1-MCP treatments) placed in a 1 m^3^ gas-tight zippered polyvinyl chloride (PVC) tent (Volcani Cube) for 1-methylcyclopropene [1-MCP, (SmartFresh™, Agrofresh, USA)] treatment at a concentration of 625 ppb (0.084 g/m^3^) [16] for 24 h at 2 ± 0.5 °C and 90 ± 5% RH. Group 2 (Control, MeJA and SA treatments) was kept in another cold room at 2 ± 0.5 °C and 90 ± 5% RH for 24 h.

### Experimental design for storage

MAPs containing all nectarine fruit, both treated and untreated with 1-MCP were closed with clips after precooling (0 ± 0.5 °C and 90 ± 5% RH for 12 h). In the study, six treatments were applied as a control, MeJA, SA, 1-MCP, MeJA + 1-MCP and SA + 1-MCP. For each treatment, 16 packages were established. The study was designed according to the completely randomized design (CRD) with four replications. The fruit were stored at 0 ± 0.5 °C and 90 ± 5% RH for 56 d. 4 packages were removed from the cold storage during each measurement period (14, 28, 42 and 56 d) for each treatment. Each of these packages represented one replicate. The packages were opened, and the fruit were stored at 20 ± 1.0 °C and 70 ± 5% RH for 2 d. Subsequently, the following measurements and analyses were conducted.

### Weight loss and firmness

The weight of nectarine fruit was recorded at regular intervals a digital precision balance (XB 12100, Presica Instruments Ltd., Switzerland) on days 0, 14, 28, 42 and 56 of storage. In addition, the weight was also recorded on two days before and after the shelf life of each measurement period. The net weight loss was calculated by summing the weight loss after both cold storage and shelf life. The results were recorded as a percentage (%).

In order to assess the firmness of flesh (20 fruit), the skin was removed from the equatorial region of the nectarine fruit. The 7.9 mm diameter tip of a fruit texture meter (GS-15, GÜSS Manufacturing Ltd., South Africa) was then immersed to a depth of 10 mm at a speed of 10 cm/min. The results were recorded as Newton (N).

### Peel color properties

The peel color of nectarine fruit was quantified by measuring CIE L*, a* and b* on both sides of the equatorial region of 20 fruit in each replicate with a colorimeter (CR-400, Konica Minolta Sensing Inc., Tokyo, Japan). Chroma (C*) and hue angle (h°) values were calculated from the a* and b* values using the formulas C*= (a*2 + b*2)^1/2^ and h°= tan^− 1^ (b*/a*), respectively [17].

### Soluble solids content and titratable acidity

Prior to analysis, the fruit were first washed with distilled water. In each replicate, a sufficient quantity of fruit was sliced with a stainless-steel knife and homogenized in a fruit shredder (Tefal, France). Subsequently, the juice was obtained by passing it through a cheesecloth. The soluble solids content (SSC) was quantified using a digital refractometer (PR-1, Atago, Japan) and the results were recorded as a percentage. Titratable acidity was determined by titrating 10 mL of juice sample with 0.1 N NaOH until the pH value reached 8.1. The quantity of NaOH consumed was used to calculate the concentration of malic acid in grams per 100 mL.

### Total phenolics and antioxidant capacity

Twenty-five milliliters of methanol were added to five grams of fruit samples from each replicate, which were then homogenized in a homogenizer (Ika Ultra-Turrax T18 Basic, Germany) for two min. Subsequently, the samples were maintained at 4 °C in the dark for 16 h, filtered through filter paper, and stored in tubes at -20 °C until analysis.

The total phenolics content of nectarine fruit was determined by a modified Folin-Ciocalteu’s method [18]. The absorbance of the prepared solutions was read at a wavelength of 725 nm in a spectrophotometer (Varian Bio 100, Australia) after 2 h in the dark at 20 °C. The results were recorded as milligrams gallic acid equivalent (GAE) per 100 g.

The antioxidant activity was quantified using the ferric reducing antioxidant power (FRAP) method, as described by Benzie and Strain [19]. The solutions prepared by the addition of the FRAP solution were maintained in the dark at 20 °C for 30 min, after which their absorbance was read at a wavelength of 593 nm in a spectrophotometer. The resulting curve was plotted in the prepared standard trolox (6-hydroxy-2,5,7,8-tetramethylchromane-2-carboxylic acid) solution, and the antioxidant capacity was calculated. The results were recorded as micromoles (µmol) of trolox equivalent (TE) per gram.

### Wooliness and acceptance

The degree of woolliness was determined by evaluating the woolliness of the fruit flesh in 20 nectarines taken from each repetition. This was defined visually, with the samples being classified into one of five groups, as follows: 0: none, 1: very little, 2: little, 3: moderate, 4: severe, 5: very severe.

The acceptability of the nectarine fruit was evaluated by five trained panelists on a 1–5 scale (1: extremely poor and soft; 2: poor or soft; 3: fair and limited marketability; 4: good; 5: excellent).

### Statistical analysis

The data obtained from the experiment were subjected to analysis of variance using the JMP statistical package program (JMP 13.2, USA). The significance of the observed differences between the means was determined by means of the Tukey test, with a significance level of 0.05.

## Results and discussion

**Fig. 1:**
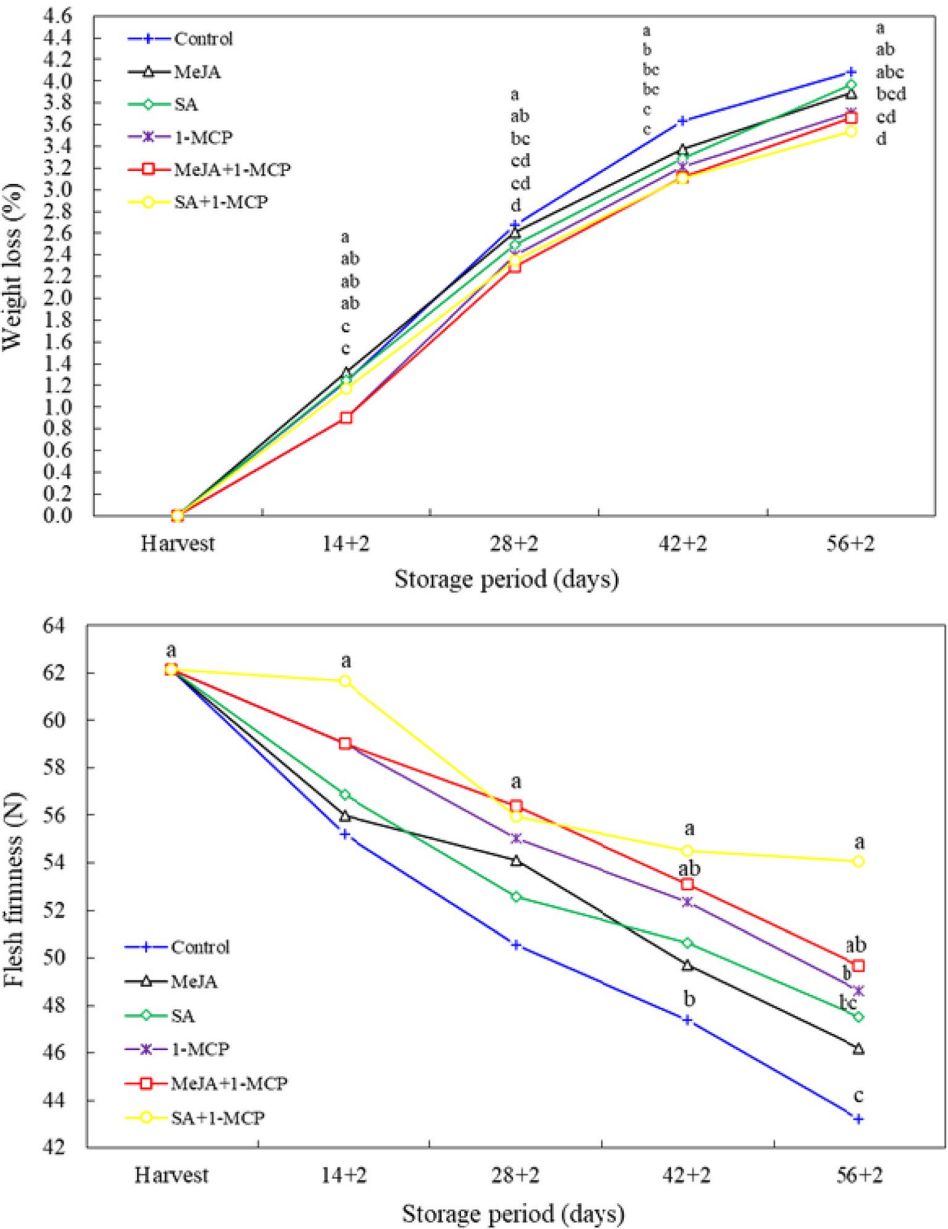
Changes in weight loss and flesh firmness of nectarine fruit treated with 1-MCP, methyl jasmonate and salicylic acid during cold storage. The difference between averages indicated by vertically different lower-case letters were significant (Tukey test, *p* ≤ 0.05)

The weight loss of MeJA + 1-MCP and SA + 1-MCP treated fruit was significantly lower than that of the control fruit at day 14 + 2. However, at day 28 + 2, the weight loss was lower than that of the control in all treatments except the MeJA treatment. While weight loss was lower in all treatments compared to the control at day 42 + 2, lower weight loss was measured only in 1-MCP, MeJA + 1-MCP and SA + 1-MCP treatments at day 56 + 2 (Fig. 1).

Following a two-day shelf-life period, all treatments exhibited a reduction in firmness. A significant (*P* < 0.05) effect of treatments was observed in the delay of firmness loss at 42 + 2 days and 56 + 2 days. At day 42 + 2, only the SA + 1-MCP-treated fruit exhibited higher firmness than the control fruit. However, at the final measurement (56 + 2 d), all the fruit treated with SA + 1-MCP, MeJA + 1-MCP and 1-MCP exhibited higher firmness than the control fruit. In the final measurement, the fruit flesh firmness of the SA + 1-MCP (54.06 N) treatment was approximately 20% higher than that of the control (43.21) (Fig. 1).

The occurrence of postharvest weight losses in fruits and vegetables results in economic losses for producers or traders. It is therefore desirable to minimize weight losses during storage and shelf life. The weight loss observed in the study ranged from 3.54 to 4.08%. In all measurement periods, the weight loss of nectarine fruit treated with 1-MCP was found to be lower than that of the control. In the last two measurement periods, the weight loss of MeJA treatment alone (3.38% and 3.89%, respectively) was also found to be lower than that of the control (3.38% and 4.08%, respectively). This may be attributed to the delaying effect of 1-MCP on ripening which has the effect of slowing down metabolic activities in fruits [20]. Indeed, it has been demonstrated that 1-MCP can delay weight loss in 1-MCP-treated peach [21], apricot [22], plum [23], nectarine [15] fruits during storage. Similarly, it has been reported that weight loss is delayed by MeJA application in plum [24, 25], persimmon, and peach [26] fruits. The weight loss retarding effect of MeJA may be attributed to its ability to maintain cellular integrity due to its anti-aging role [27, 28].

In the context of the market, consumer demand for fruit that is overly soft is relatively low. Concurrently, it is advantageous to extend the period of fruit softening in order to achieve a longer shelf life. During the study, a reduction in firmness was observed in the fruit of all treatments. However, in the final two measurement periods, the firmness of the fruit treated with 1-MCP, MeJA and SA was found to be 7.0–25.0% higher than that of the control group fruit. Consequently, all growth regulator treatments resulted in a delay in fruit softening. Indeed, Özkaya et al. [5] reported that the flesh firmness of nectarine fruit treated with 1-MCP was higher than the control. The observed slowing of the loss of fruit flesh firmness by growth regulators (1-MCP, MeJA and SA) may be attributed to their ripening-delaying effects [5.11,14,26,27].

**Fig. 2:**
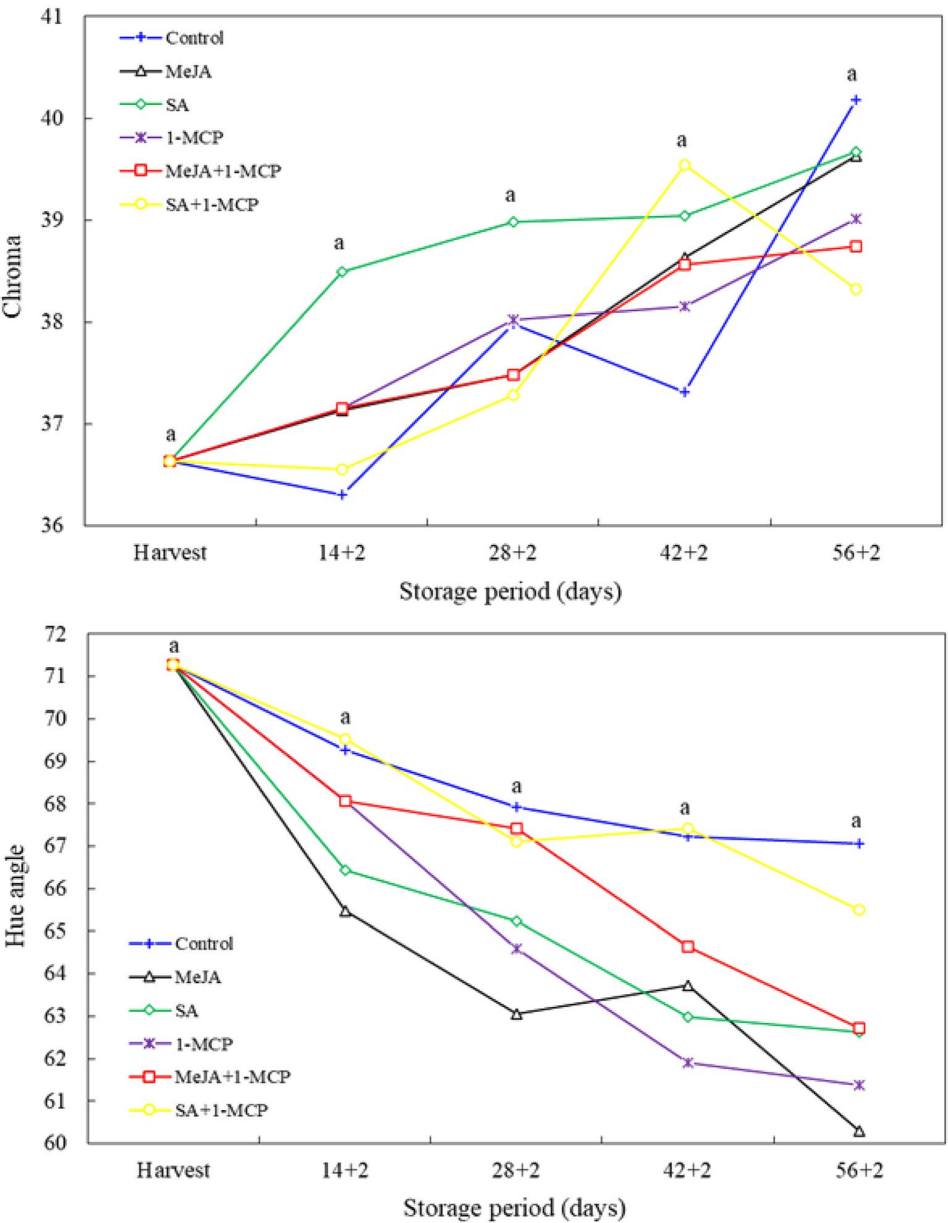
Changes in chroma and hue angle of nectarine fruit treated with 1-MCP, methyl jasmonate and salicylic acid during cold storage. The difference between averages indicated by vertically different lower-case letters were significant (Tukey test, *p* ≤ 0.05)

In general, chroma values increased with the progression of the storage period, whereas hue angle values decreased. However, the effect of the treatments was found to be insignificant in all measurement periods (Fig. 2). Thusly, research findings indicate that applications of growth regulators such as 1-MCP, MeJA and SA have no discernible impact on fruit color traits. No effect was observed on color traits when MeJA was applied to plum fruit [2], 1-MCP to banana fruit [29] and SA to peach fruit [30]. However, there are also research findings indicating that growth regulators studied may influence color traits. Aslantürk et al. [31] reported that MeJA affected fruit color in apricot, Ezzat et al. [27] reported that SA affected fruit color in apricot, and Wang et al. [21] recorded that 1-MCP affected fruit color in peach fruit.

**Fig. 3:**
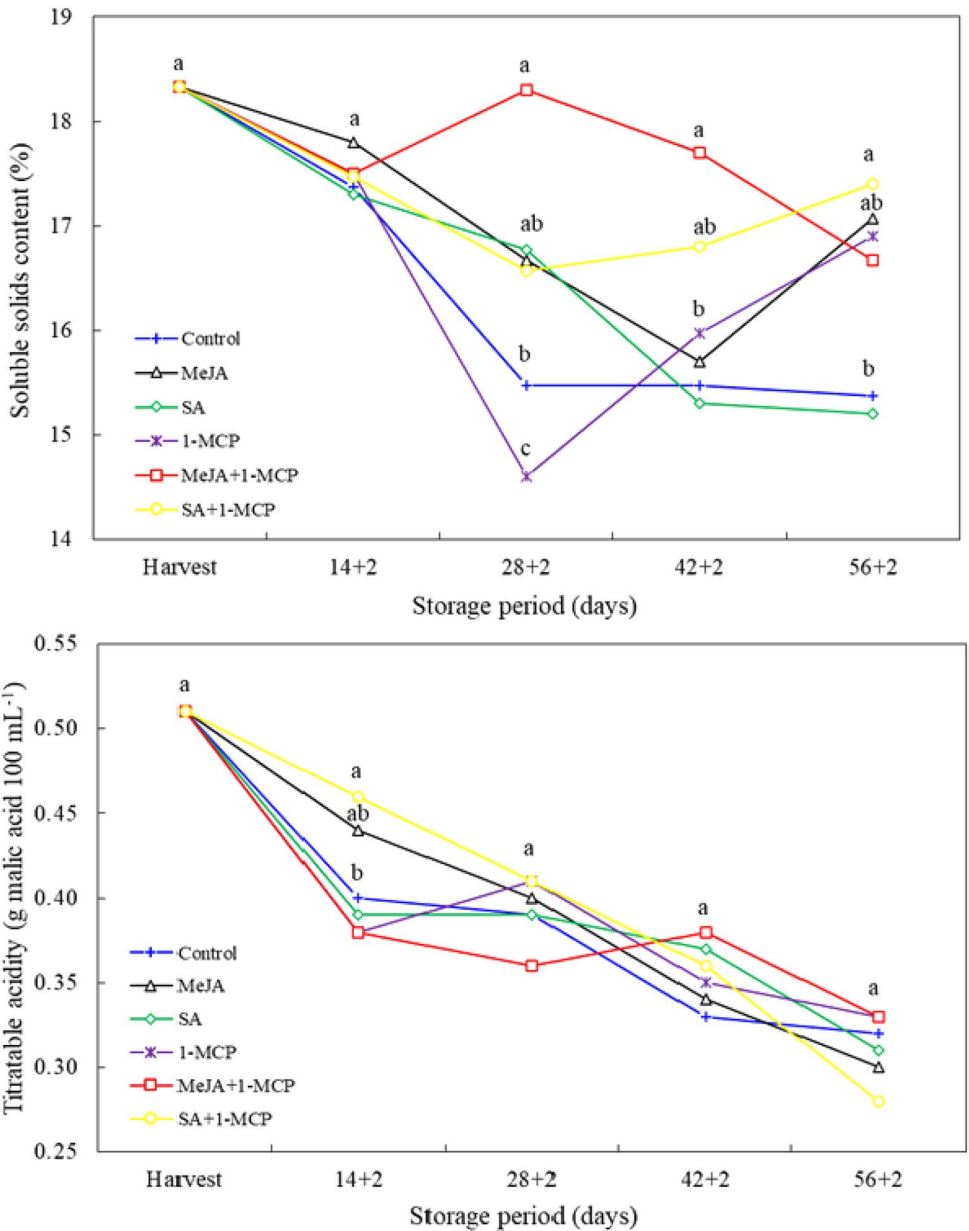
Changes in soluble solids content and titratable acidity of nectarine fruit treated with 1-MCP, methyl jasmonate and salicylic acid during cold storage. The difference between averages indicated by vertically different lower-case letters were significant (Tukey test, *p* ≤ 0.05)

In the three most recent measurement periods, the impact of the treatments on the soluble solids content (SSC) was statistically significant (*p* < 0.05). At 28 + 2 days, the SSC of MeJA + 1-MCP-treated fruit was found to be higher than that of the control, whereas the SSC of 1-MCP-treated fruit was found to be lower. Similarly, at day 42 + 2, only the MeJA + 1-MCP treatment exhibited a higher SSC than the control. At the final measurement, only the fruit treated with SA and 1-MCP exhibited a higher SSC than the control. With regard to titratable acidity, the treatments exhibited significant (*P* < 0.05) differences from one another only on day 14 + 2. During this period, only the SA + 1-MCP treatment (0.46% malic acid) exhibited a higher acidity content than the control (0.40% malic acid) (Fig. 3).

Consumer preferences may vary according to the SSC and acid content of the fruit. The perception of fruit taste is influenced by the concentration of SSC and the level of astringent acidity [32]. In the present study, the higher SSC observed in the fruit of the 1-MCP treatments combined with MeJA and SA compared to the control was statistically significant. Baswal et al. [11] reported that MeJA and SA treatments in ‘Kinnow’ mandarin increased the SSC content, while Kucuker and Ozturk [24] reported that a MeJA treatment in plum increased the SSC content. However, in the present study, the acidity level of the fruit was found to be similar to that of the control fruit during storage. In previous studies, no effect of growth regulator applications on acidity in apricot [31] and plum [8] was recorded. The discrepancy in the impact of growth regulator applications on SSC and acidity can be attributed to the variations in species, cultivar, application dosage, and timing preferred across the studies [10].

**Fig. 4:**
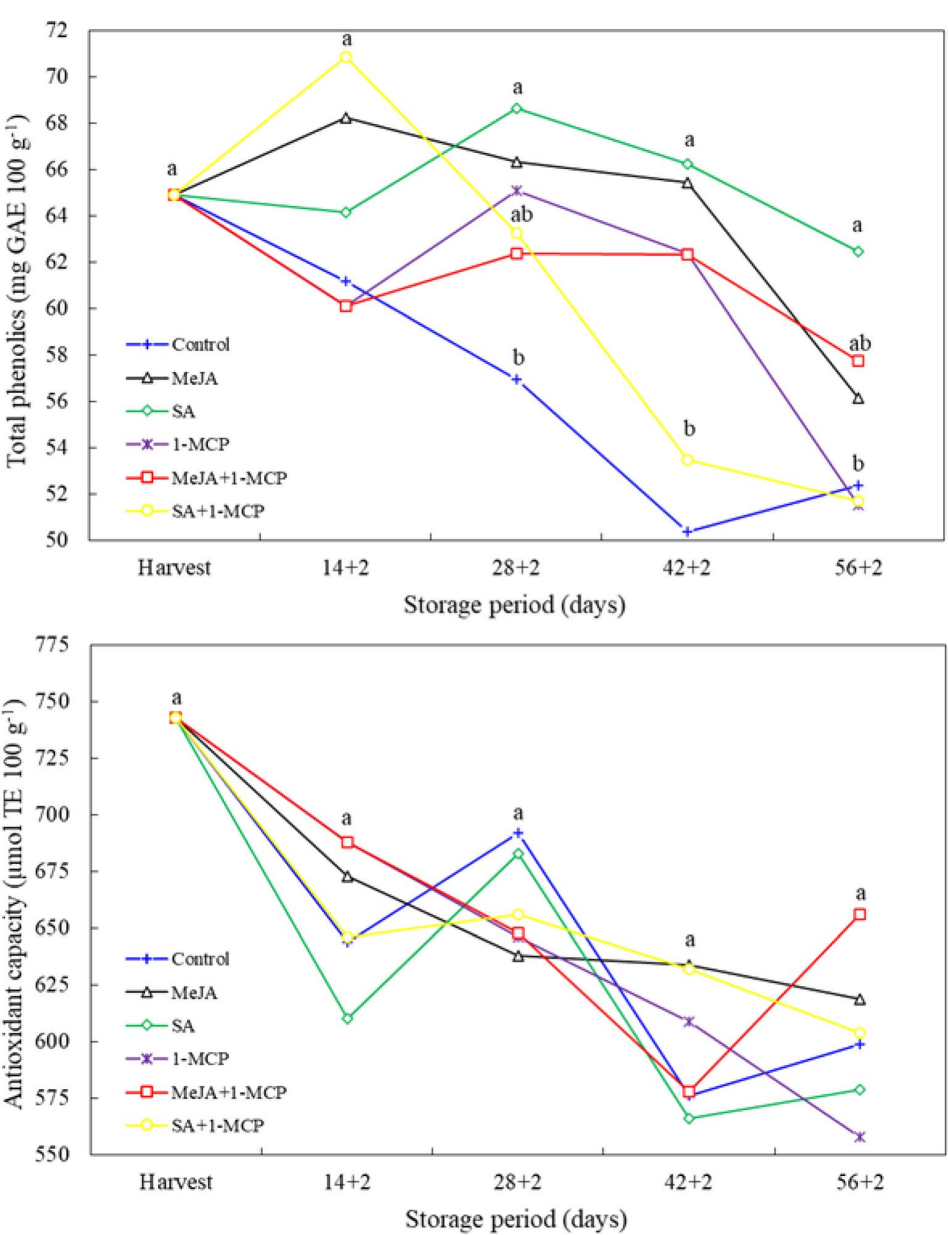
Changes in total phenolics and antioxidant capacity of nectarine fruit treated with 1-MCP, methyl jasmonate and salicylic acid during cold storage. The difference between averages indicated by vertically different lower-case letters were significant (Tukey test, *p* ≤ 0.05)

In the three most recent measurement periods, the impact of the treatments on total phenolics was statistically significant (*P* < 0.05). At 28 + 2 days, total phenolics were higher than the control in the SA, MeJA and 1-MCP treatments; at 42 + 2 days, in all treatments except SA + 1-MCP; and in the last measurement period, only in the SA treatment. In the final measurement period, the antioxidant capacity of nectarines exhibited a decline in comparison to the harvest period. Nevertheless, during the measurement periods, the antioxidant capacity of fruit treated with growth regulators was comparable to that of the control (Fig. 4).

Phenolic compounds are secondary metabolites that act as natural antioxidants in plants, reducing the damage caused by free radicals to cells by reducing oxidative stress and having a therapeutic role in diseases [33]. Furthermore, it plays a role in the development of sensory properties, such as color, taste and flavor, of the fruit. Additionally, it increases its resistance to stress and diseases [34]. The results of our study indicated that the application of growth regulators had a significant impact on the total phenolic content of nectarine fruit. In particular, the total phenolics of the SA and MeJA-treated fruit were found to be higher than those of the control fruit. Similarly, Baek et al. [33] observed that tomato fruit treated with MeJA and SA exhibited higher total phenolics than control fruit. Furthermore, Aslantürk et al. [31] observed a higher total phenolic content in MeJA-treated apricot fruit, while Tareen et al. [35] reported a higher total phenolic content in SA-treated peach fruit compared to the control group. In the present study, no effect of the treatments on antioxidant activity was observed. Similarly, Karaman et al. [2] reported that MeJA had no effect on antioxidant activity in plum, Alrashdi et al. [36] reported that salicylic acid had no effect on table grape, and Fawbush et al. [37] reported that 1-MCP had no effect on antioxidant activity in apple fruit. Additionally, research reports have been published indicating the opposite [33, 38].

**Fig. 5:**
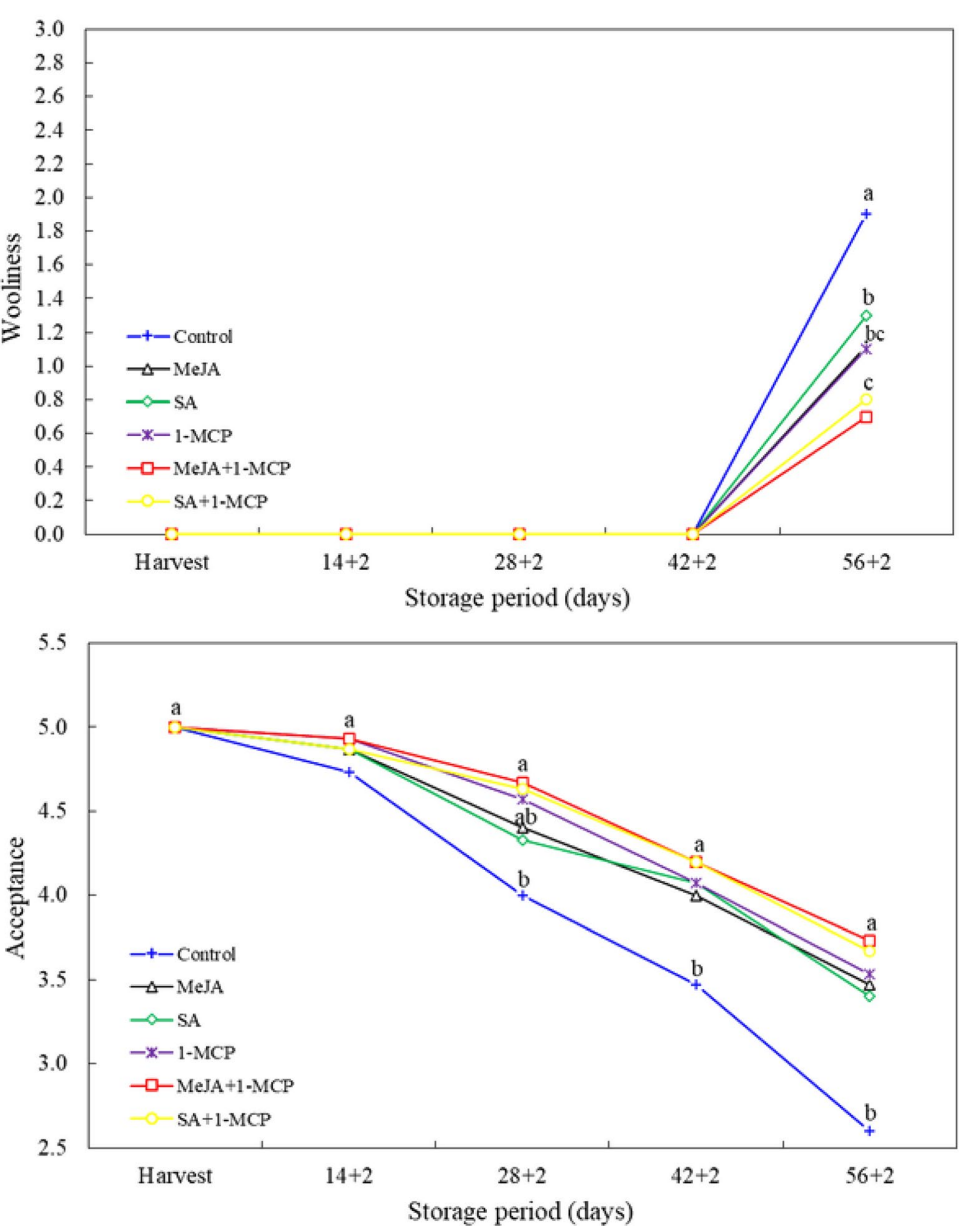
Changes in wooliness and acceptance of nectarine fruit treated with 1-MCP, methyl jasmonate and salicylic acid during cold storage. The difference between averages indicated by vertically different lower-case letters were significant (Tukey test, *p* ≤ 0.05)

With the exception of the final measurement period (56 + 2 d), no wooliness was observed in nectarine fruit during the other measurement periods. The wooliness of the fruit was found to be significantly lower in those treated with growth regulators (SA, MeJA and 1-MCP) in comparison to the control. Concomitantly, wooliness was observed to be lower in fruit of the MeJA + 1-MCP and SA + 1-MCP treatments in comparison to the SA treatment. In terms of consumer preference, fruit treated with growth regulators were perceived as more acceptable in the last two measurement periods (42 + 2 and 56 + 2 d). However, at 28 + 2 d, consumer acceptance of all 1-MCP-treated fruit was found to be higher than that of the control and the other treatments (SA and MeJA) (Fig. 5).

Sensory characteristics play an important role in consumer acceptance of fruits and vegetables [11]. Therefore, it is advantageous to have a low wooliness in nectarines, which reduces consumer acceptance. In the present study, the wooliness of nectarine fruit treated with growth regulators was found to be significantly reduced. In particular, the wooliness of the fruit was found to be lower in those treated with 1-MCP in combination with other treatments. Consequently, the level of acceptance was found to be higher. The maturity-retarding effect of 1-MCP may have slowed down the aging of nectarine fruit, consequently delaying the occurrence of wooliness. Indeed, wooliness of nectarine fruit can occur as a result of prolonged storage at low temperatures [20].

## Conclusion

After cold storage, nectarines experience quality losses during shelf life. In particular, irreversible flavor loss occurs during this process and ethylene biosynthesis increases [39]. In the study, the single treatments of MeJA, SA and 1-MCP, and combined treatments of 1-MCP, were found to contribute to the maintenance of fruit quality characteristics of the ‘Extreme Sunny’ nectarine cultivar during shelf life. The treatments with 1-MCP were effective in delaying weight loss and softening of nectarine fruit, while the MeJA and SA treatments were effective in maintaining quality by limiting the loss of total phenolics. In conclusion, the results of this study demonstrate that fruit treated with MeJA and 1-MCP, and SA and 1-MCP, respectively, can be successfully stored for 2 days, in addition to 56 days, without any significant deterioration in quality. 1-MCP can be used as an effective tool to maintain nectarine fruit quality over a longer period of time by inhibiting ethylene production in the fruit.

## Data Availability

Data will be available on request to corresponding authors.

## Abbreviations

1-MCP: 1-methylcyclopropene
C*: Chroma
CIE: Commission de International de I’Eclairage
FRAP: Ferric Reducing Antioxidant Power
GAE: Gallic acid equivalent
h: Hour
h°: Hue angle
m: Meter
MAP: Modified atmosphere packaging
MeJA: Methyl jasmonate
min: Minute
mL: Milliliter
mM: Millimolar
N: Normality
NaOH: Sodium hydroxide
pH: Power of hydrogen
PVC: Polyvinyl chloride
SA: Salicylic acid
SSC: Soluble solids content
TE: Trolox equivalent
